# Restoring Oral Hygiene Autonomy Through a Customized Toothbrush Handle in a Patient With Rheumatoid Arthritis and Fibromyalgia

**DOI:** 10.1002/ccr3.72276

**Published:** 2026-03-12

**Authors:** Khalid Alfaifi, Ali Robaian, Abdulaziz alsakr, Wael Alanazi, Abdullah Saad Alqahtani

**Affiliations:** ^1^ Saudi Board of Family Dentistry, College of Dentistry Prince Sattam Bin Abdulaziz University Al‐Kharj Saudi Arabia; ^2^ Conservative Dental Sciences Department College of Dentistry Prince Sattam Bin Abdulaziz University Alkharj Saudi Arabia; ^3^ Department of Preventive Dental Sciences, College of Dentistry Prince Sattam Bin Abdulaziz University Alkharj Saudi Arabia; ^4^ Department of Preventive Dental Sciences, College of Dentistry Prince Sattam Bin Abdulaziz University Al‐kharj Saudi Arabia

**Keywords:** disability and oral health, ergonomic handle, fibromyalgia, oral hygiene, plaque index, rheumatoid arthritis, toothbrush modification

## Abstract

Early identification of functional limitations in patients with rheumatoid arthritis (RA) and fibromyalgia (FM) is essential, as chronic joint pain and reduced dexterity often impair basic oral hygiene practices. This case highlights a simple, cost‐effective, and reproducible intervention using a customized soft putty toothbrush handle fabricated chairside and molded directly to the patient's grip. A 27‐year‐old male with longstanding RA and FM, and a history of smoking, presented with poor plaque control and brushing‐associated pain. Following the chairside fabrication of an adaptive handle individually shaped to his grasp, plaque index improved dramatically from 91% to 15% within four months, with a corresponding reduction in discomfort from a VAS score of 5 to 1. This personalized ergonomic solution significantly improved brushing efficiency, comfort, and self‐care independence, underscoring the importance of tailored oral hygiene aids in medically compromised populations.

## Key Clinical Message

Customized toothbrush handles offer a practical, low‐cost solution to restore oral hygiene autonomy in patients with RA and FM. This ergonomic adaptation significantly improves plaque control and reduces discomfort, emphasizing the value of personalized dental aids in enhancing daily self‐care and quality of life for medically compromised individuals.

## 1 Introduction

A toothbrush is a fundamental tool for maintaining oral hygiene. Insufficient cleaning of the teeth enables bacteria to interact with food particles, leading to the production of lactic acid, which can cause tooth decay [1]. In addition, dental plaque is an oral bacterial biofilm that continuously accumulates on the surfaces of teeth. This plaque may play an important role in oral health or the development of diseases such as gingivitis and periodontitis if not effectively removed, plays a key role in the development of gingivitis and periodontitis [2].

However, individuals with musculoskeletal disorders such as rheumatoid arthritis (RA) and fibromyalgia (FM) often experience significant challenges in performing basic oral care tasks, including daily toothbrushing, due to reduced manual dexterity, chronic pain, and fatigue.

RA is a chronic autoimmune disorder that causes inflammation in the synovial joints. It frequently results in deformity of the hands and fingers, leading to stiffness and loss of function. Involvement of the small joints causes poor grasp and limited range of motion, making the use of oral hygiene instruments such as toothbrushes particularly difficult [3, 4].

FM, which is associated with chronic pain, is characterized by widespread musculoskeletal discomfort and fatigue as well as cognitive changes. It reduces physical and mental capacities and can lower motivation for basic tasks like daily oral care [5]. Several oral complaints, including xerostomia, glossodynia, and dysgeusia, have been reported in patients with FM [6]. Dry mouth, also referred to as xerostomia, is a subjective feeling of dryness that frequently coexists with hypofunction of the salivary glands. The incidence of xerostomia in FM patients varies between 7% and 71% [7], indicating a substantial burden irrespective of xerogenic medication use. Xerostomia is associated with an increased prevalence of caries, periodontal disease, dysphagia, oral ulcers, and candidiasis. Therefore, appropriate management of xerostomia is essential to prevent oral complications [8].

Smoking further compounds these challenges, as a well‐known major risk factor for periodontal disease because of its ability to impair healing and increase inflammation and plaque accumulation [9]. When combined with physical and functional limitations, smoking significantly increases the difficulty of maintaining adequate oral hygiene and accelerates disease progression in medically compromised patients.

As a result, poor oral hygiene is commonly observed in this population and is linked to an increased risk of periodontal disease and dental caries [10, 11]. In addition, periodontal inflammation may exacerbate systemic inflammatory conditions such as RA and FM, highlighting the bidirectional relationship between oral and systemic health [12, 13].

To monitor and quantify oral hygiene status, the O'Leary Plaque Control Record is frequently used in clinical settings to assess the percentage of plaque‐covered tooth surfaces [14]. Pain associated with oral hygiene tasks, particularly in patients with hand dysfunction, can be reliably assessed using the Visual Analog Scale (VAS), a validated tool for measuring discomfort during activities such as toothbrushing [15].

Considering such challenges, it is paramount for dental professionals to identify underlying issues and provide specific strategies to enhance oral hygiene results. One such strategy involves modifying the design of oral hygiene tools, such as toothbrush handles. These adaptations have been shown to improve compliance, reduce discomfort, and enhance brushing effectiveness in patients with restricted manual dexterity [16, 17].

A recently published study used condensation silicone impression material to mold the hands of patients with Down syndrome to customize toothbrush handles and reported significant improvements in daily plaque control compared with conventional toothbrushes [18].

There have been many ideas for adaptive aids to help patients with limited hand function; however, evidence remains limited regarding toothbrush handle modifications for individuals with RA and FM. RA makes joints stiff and deformed, while FM causes widespread pain and fatigue, both of which interfere with consistent oral hygiene practices.

Furthermore, limited data are available on the impact of such modifications on clinical oral health outcomes and patient comfort in routine practice. This case report therefore aims to evaluate the effectiveness of a low‐cost toothbrush handle modification in improving oral hygiene and reducing brushing‐associated discomfort in a patient with RA and FM.

## 2 Case History/Examination

A 27‐year‐old male with a 10‐year history of RA and FM, and an active smoking habit, presented to the dental clinic complaining of generalized tooth hypersensitivity. His current medications included Pregabalin (150 mg), Hydroxychloroquine Sulfate (200 mg), and Amitriptyline (10 mg), prescribed by his rheumatologist.

The patient reported significant joint stiffness and muscular fatigue, particularly affecting hand function, which impaired his ability to maintain effective oral hygiene. Clinical examination revealed heavy plaque accumulation and cervical carious lesions involving more than 21 teeth. Signs of xerostomia were also noted.

Prior to initiating treatment, the patient was thoroughly briefed on the proposed procedures, and written informed consent was obtained. To ensure consistency, all clinical assessments were performed by the same calibrated examiner.

## 3 Methods (Investigations, and Treatment)

Initial assessment involved evaluating dental plaque using the O'Leary Plaque Index. The patient was instructed to chew two disclosing tablets—one per side—for 30 s, followed by swishing and expectoration. The resulting plaque index was 91%, indicating severe plaque accumulation. During the second visit, the patient brought his personal toothbrush. He was instructed to simulate his usual brushing technique, during which discomfort was measured using the Visual Analog Scale (VAS). He reported a pain score of 5 out of 10, confirming moderate brushing‐related discomfort.

To address the patient's limited manual dexterity, a custom ergonomic toothbrush handle was fabricated chairside using a soft putty impression material (3 M Express XT Putty Soft VPS): The putty was applied around the entire toothbrush handle—from the neck near the brush head to the base. The patient was asked to grip the handle and squeeze gently until experiencing mild joint tension without pain. The material was allowed to set completely (approximately 3 min and 30 s), forming a handle customized to the patient's natural grip.

This personalized adaptation minimized the need for finger flexion and reduced grip pressure, thereby enhancing comfort and usability during toothbrushing.

## 4 Conclusion and Results (Outcome and Follow‐Up)

**FIGURE 1:**
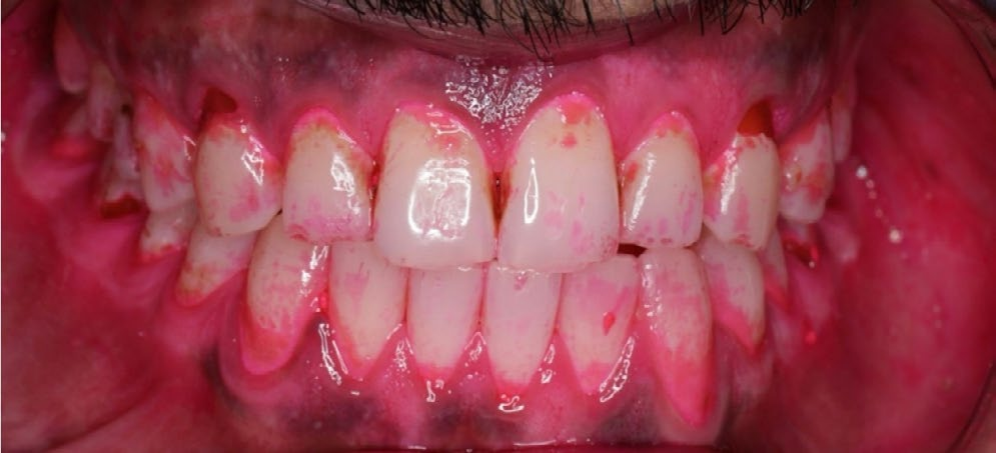
Description: Baseline disclosing solution image showing heavy plaque accumulation before toothbrush handle modification (Plaque Index: 91%).

Following the fabrication of the customized toothbrush handle, the patient was instructed to simulate toothbrushing once again to reassess discomfort levels. He reported a Visual Analog Scale (VAS) score of 1 out of 10, demonstrating a marked reduction in pain compared to the initial score of 5 out of 10 when using his conventional toothbrush (Figure 1).

Post‐intervention, the patient was educated on effective brushing techniques and advised to brush twice daily using a high‐fluoride toothpaste. The patient was advised to use xylitol‐containing chewing gum with a total daily xylitol intake of approximately 5–10 g/day, divided into multiple short sessions (e.g., after meals, 3–5 times daily, 10–20 min per session). The selected dosage range (5–10 g/day) is supported by established literature demonstrating that caries‐preventive effects of xylitol are dose‐dependent, with significant benefit observed at daily exposures of approximately 5–7 g or higher [19].

**FIGURE 2:**
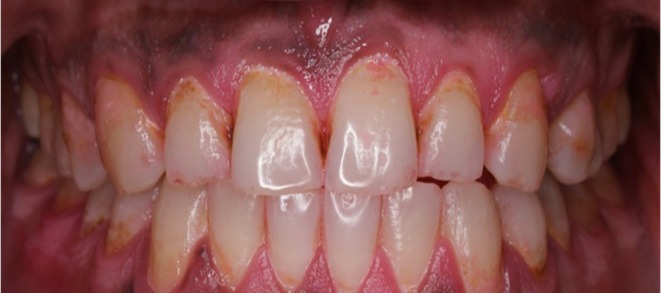
After 4 months, post‐intervention image demonstrating a significant reduction in dental plaque following four months of using the customized toothbrush handle (final Plaque Index: 15%).

At the four‐month follow‐up, the patient reported significant improvements in grip control and brushing efficiency. Clinical reassessment showed a dramatic reduction in plaque index from 91% to 15% with no signs of gingival inflammation or new carious lesions (Figure 2).

**FIGURE 3:**
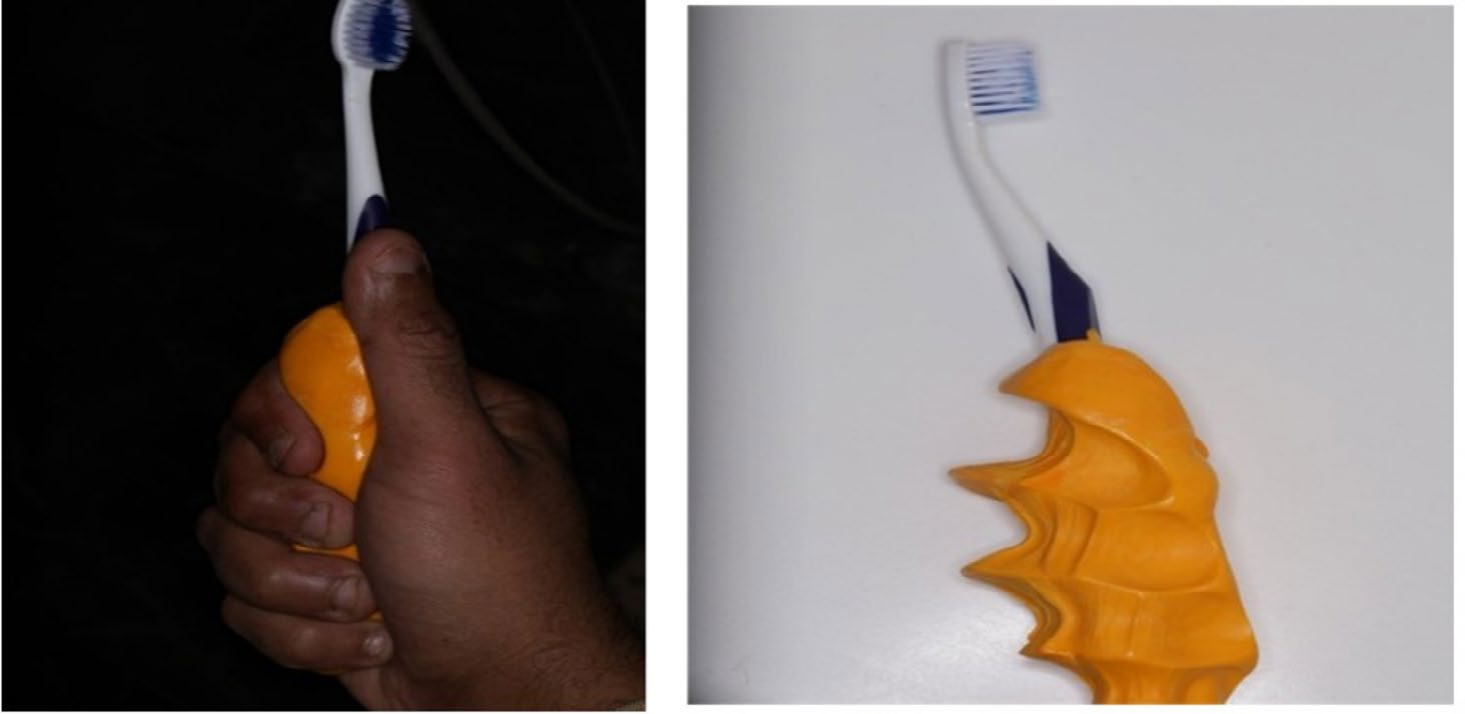
Fabrication of toothbrush handle: Molding of participant's hand using 3 M Express XT Putty Soft VPS Impression Material.

The intervention was well‐tolerated, and no additional oral hygiene aids or medications were introduced throughout the follow‐up period. These findings suggest that the customized toothbrush handle was the primary contributor to the patient's clinical improvement (Figure 3). The patient also experienced a notable boost in confidence and independence in performing daily oral care tasks. This case underscores the effectiveness of simple, cost‐efficient, patient‐centered ergonomic solutions in addressing oral hygiene challenges faced by individuals with chronic musculoskeletal disorders. A minor chairside modification, requiring minimal time and resources, resulted in an 84% improvement in plaque control, enhanced comfort, and restored autonomy. Such tailored interventions should be routinely considered by dental practitioners, especially when managing medically compromised patients with reduced manual dexterity. Personalized adaptive aids can play a pivotal role in improving adherence, self‐care, and overall quality of life.

## 5 Discussion

Patients with RA and FM often experience substantial difficulties in maintaining adequate oral hygiene due to joint pain, reduced grip strength, and persistent fatigue [5, 20]. These physical limitations compromise effective plaque removal, thereby increasing the risk of periodontal disease and dental caries, conditions that may further contribute to systemic inflammation and negatively affect overall quality of life [12]. In the present case, the patient, who was also an active smoker, exhibited a high plaque index of 91%, reflecting inadequate plaque control. Smoking is a well‐established risk factor for periodontal disease due to its detrimental effects on immune response and tissue healing [21]. When combined with RA and FM‐related functional impairments, the ability to perform effective oral hygiene measures becomes particularly challenging. This observation is consistent with previous reports indicating higher plaque accumulation and poorer oral health outcomes among RA and FM patients compared with healthy individuals [22, 23].

To address these barriers, we implemented a simple, cost‐effective solution: a chairside‐fabricated, patient‐molded toothbrush handle using soft putty material. The intervention resulted in a dramatic 84% reduction in plaque index and an 80% reduction in brushing discomfort (VAS) over four months. These improvements support prior studies advocating for adaptive oral hygiene devices in patients with manual dexterity limitations [1, 17, 24, 25]. A structured comparison between these studies and the current intervention is presented in Table 1.

**TABLE 1:** presents a comparative summary of previously published studies and the present case, illustrating that the current intervention achieved the greatest improvement in plaque reduction and brushing comfort through a rapid, low‐cost chairside fabrication technique.

Study/Year	Population/Condition	Modification method	Outcome	Remarks
Droubi et al. 2021 [18]	Children with Down syndrome (*n*≈30; 8–12 years)	Customized silicone‐molded toothbrush handles versus conventional	Significant reduction in plaque index (*p* < 0.001)	Demonstrated feasibility and plaque removal improvement in pediatric population
Yuen et al. 2011 [17]	Adults with systemic sclerosis	Multifaceted oral hygiene intervention using an ergonomic handle and education	Improved gingival health and plaque control (*p* < 0.05)	Combined ergonomic and behavioral approach improved outcomes
Colvenkar et al. 2022 [23]	Adults with limited manual dexterity	Individually modeled 3D‐printed toothbrush handle	Better plaque removal and grip comfort	High‐cost, laboratory‐dependent method
Present Case	Rheumatoid arthritis + Fibromyalgia	Chairside‐fabricated ergonomic handle molded from 3 M Express XT Putty Soft VPS	Plaque ↓ 91% → 15%; VAS pain ↓ 5 → 1	Low‐cost, rapid chairside fabrication achieving quantifiable improvement in plaque control and brushing comfort

While previous efforts have focused on 3D‐printed designs or special‐needs populations like children with Down syndrome [18] or adults with systemic sclerosis [17], this case uniquely documents the successful use of a chairside‐molded adaptive handle in a patient with dual autoimmune conditions (RA and FM). Compared to high‐cost or lab‐based solutions [24, 25, 26], our method is affordable (under $2), rapid (under 5 min), and reproducible in any general dental setting.

This case is one of the few documented reports to show quantifiable improvement in both plaque control and brushing comfort in a medically complex patient using a low‐cost, chairside‐fabricated toothbrush handle. Its simplicity, patient‐centered customization, and clinical effectiveness make it well‐suited for routine use, especially in resource‐limited settings. The model is scalable and offers a replicable framework for improving oral hygiene autonomy in patients with neuromuscular or autoimmune impairments.

In addition to mechanical benefits, such interventions may foster patient autonomy, adherence, and confidence, psychological factors critical for sustained self‐care [27]. Improving oral hygiene may also reduce systemic inflammation, considering the bidirectional relationship between periodontal and autoimmune diseases like RA and FM [13, 28].

The patient's xerostomia, a common symptom in FM with a prevalence of up to 71% [8], was addressed through xylitol gum. Evidence from meta‐analyses supports this as a non‐pharmacological method to stimulate salivary flow and reduce caries risk [29, 30].

This case highlights the importance of interdisciplinary, patient‐centered care. A simple ergonomic modification substantially improved oral hygiene, comfort, and independence without specialized tools or lab equipment, making it viable for widespread clinical application.

Beyond dental management, optimal oral health outcomes in patients with RA and FM require collaboration across multiple healthcare disciplines. Rheumatologists play a central role in controlling systemic inflammation and joint disease activity, which directly influences hand function and the patient's ability to perform oral self‐care. Occupational therapists are uniquely positioned to assess limitations in grip strength, dexterity, and endurance, and to recommend adaptive strategies or assistive devices that complement chairside dental modifications. Dental hygienists contribute through individualized oral hygiene instruction, reinforcement of adaptive techniques, and ongoing monitoring of plaque control, while primary care physicians support the management of contributory factors such as smoking cessation, medication side effects, fatigue, and xerostomia. In addition, behavioral health professionals may assist patients in addressing chronic pain, anxiety, and motivational barriers that affect adherence to daily self‐care. Integrating these disciplines supports functional independence and aligns with holistic models of care for medically complex patients [10, 31]. Dentists who feel uncomfortable performing chairside adaptations themselves, or in cases where more complex modifications are required, may appropriately refer patients to occupational therapy for skilled assessment and intervention in this area [32].

However, this study is limited by its single‐patient design. The observed outcomes may partially reflect increased motivation following tailored instruction. Furthermore, no quantitative measurements of hand strength or dexterity were performed, and the follow‐up period was limited to four months, restricting long‐term conclusions.

Future research should involve larger cohorts with varying degrees of impairment, longer follow‐up periods, and standardized tools to assess grip strength, usability, and patient satisfaction. Exploring alternative materials, handle shapes, and quick‐modification techniques could further improve adaptability and comfort across diverse patient populations.

## Author Contributions

**Khalid Alfaifi:** conceptualization, data curation, investigation, writing – original draft. **Ali Robaian:** conceptualization, investigation, project administration, supervision. **Abdulaziz alsakr:** conceptualization, formal analysis, investigation, validation. **Wael Alanazi:** investigation, project administration, validation, writing – original draft. **Abdullah Saad Alqahtani:** conceptualization, data curation, investigation, supervision, writing – original draft, writing – review and editing.

## Funding

The authors have nothing to report.

## Consent

Written informed consent was obtained from the patient to publish this report in accordance with the journal's patient consent policy.

## Conflicts of Interest

The authors declare no conflicts of interest.

## Data Availability

The data that support the findings of this study are available on request from the corresponding author.
